# Associations between Vitamin D Deficiency and Carbohydrate Intake and Dietary Factors in Taiwanese Pregnant Women

**DOI:** 10.3390/medicina59010107

**Published:** 2023-01-03

**Authors:** Chao-Hsu Lin, Pei-Shun Lin, Meei-Shyuan Lee, Chien-Yu Lin, Yi-Hsiang Sung, Sung-Tse Li, Shun-Long Weng, Shing-Jyh Chang, Hung-Chang Lee, Yann-Jinn Lee, Hung-Yang Chang, Chih-Sheng Lin

**Affiliations:** 1 Department of Pediatrics, Hsinchu MacKay Memorial Hospital, Hsinchu 300, Taiwan; 2 Department of Biological Science and Technology, National Yang Ming Chiao Tung University, Hsinchu 300, Taiwan; 3 Department of Medicine, MacKay Medical College, New Taipei 252, Taiwan; 4 Department of Nutrition, Hsinchu MacKay Memorial Hospital, Hsinchu 300, Taiwan; 5 Department of Health Care Management, National Taipei University of Nursing and Health Sciences, Taipei 112, Taiwan; 6 School of Public Health, National Defense Medical Center, Taipei 114, Taiwan; 7 Department of Obstetrics and Gynecology, Hsinchu MacKay Memorial Hospital, Hsinchu 300, Taiwan; 8 Department of Pediatrics, MacKay Children’s Hospital, Taipei 104, Taiwan; 9 Department of Medical Research, Tamshui MacKay Memorial Hospital, New Taipei 251, Taiwan; 10 Department of Pediatrics, School of Medicine, College of Medicine, Taipei Medical University, Taipei 110, Taiwan; 11 Institute of Biomedical Sciences, MacKay Medical College, New Taipei 252, Taiwan; 12 Center for Intelligent Drug Systems and Smart Bio-Devices (IDS2B), National Yang Ming Chiao Tung University, Hsinchu 300, Taiwan

**Keywords:** vitamin D, vitamin D supplementation, pregnant women, carbohydrate

## Abstract

This cross-sectional observation study investigated the vitamin D (VD) status in Taiwanese pregnant women and the effects of VD supplementation and macronutrient intake on serum 25-hydroxy-vitamin D (25[OH]D) level. Data on VD intake, daily sunlight exposure, and carbohydrate intake were obtained from 125 pregnant women at 30–37 weeks’ gestation. Serum 25[OH]D level was measured before delivery in all enrolled women; and the mean 25(OH)D level was 43 nmol/L or 17.2 ng/mL. The 25(OH)D level was significantly correlated with total VD intake of pregnant women (r = 0.239; *p* = 0.007). The severe VD deficiency group (*n* = 16; mean of 25(OH)D level = 8.5 ng/mL) had significantly lower total VD intake and supplementation than the groups with VD deficiency (*n* = 69), insufficiency (*n* = 32), and sufficiency (*n* = 8). Those with ≥400 IU/day total VD intake (including VD from food and supplementation) had significantly higher 25(OH)D concentration than those with <400 IU/day total VD intake. Those with 400 IU/day VD supplementation could significantly increase serum 25(OH)D concentrations for pregnant women. Among 85 pregnant women with carbohydrate intake of ≥300 g/day, serum 25(OH)D levels were negatively correlated with carbohydrate intake (*p* = 0.031). In conclusion, VD deficiency was highly prevalent in Taiwanese pregnant women. VD supplementation was the most effective method for increasing 25(OH)D concentration in pregnant women. Higher carbohydrate intake might reduce 25(OH)D levels.

## 1. Introduction

Vitamin D (VD) is a fat-soluble steroid hormone responsible for maintaining calcium and phosphate homeostasis as well as bone mineral metabolism [1]. Moreover, VD influences more than 200 genes, which are responsible for the regulation of cellular proliferation, differentiation, apoptosis, gene expression, angiogenesis, and immune modulation [2,3]. Hence, VD deficiency may be associated with several chronic illnesses, including cancers, hypertension, osteoarthritis, diabetes, autoimmune diseases, infectious diseases, asthma, leaky gut, pain, and cardiovascular diseases [2,4,5,6]. Pregnant women are at a risk for developing VD deficiency. Fetus develops collagen matrix for its skeleton during the 1st and 2nd trimesters, and calcifies the skeleton in the 3rd trimester; therefore, the maternal demand for calcium and VD during pregnancy should be increased [7]. Furthermore, VD deficiency increases the risk of preeclampsia, cesarean section, gestational diabetes, and preterm birth in pregnant women [7,8,9,10]. Moreover, pregnant women with VD deficiency are at an increased risk for low birth weight, hypocalcemia, delay in fetal lung and bone development, respiratory tract infection, wheezing, central nervous system disorder, and type 1 diabetes in offspring [2,8,10,11]. Maternal VD levels are also highly correlated with infant VD levels at birth [12,13,14]. In brief, VD is important for both pregnant women and neonates. An estimate of the economic burden of VD deficiency in pregnant women in the United Kingdom has been reported. Addressing VD inadequacy in pregnant women in England and Wales would reduce the number of cases of preeclampsia by 4126, resulting in a net saving of £18.6 million for the National Health Service in England and Wales [15].

Dietary factors also contribute to vitamin D status during pregnancy [16]. However, few studies have addressed the relationship between Vitamin D and carbohydrate intake. Nutrigenetic studies in Indonesia demonstrated that the relationships between vitamin D, body fat percentage and newborn anthropometry was modified by high carbohydrate intake (mean 405 g/day and 319 g/day carbohydrate intake) [17,18]. But there was no exact cut-off value of high carbohydrate intake in these studies. During pregnancy, Institute of Medicine (IOM; U.S.) and Taiwan’s Dietary Reference Intakes recommended that the estimated average requirement (EAR) for dietary carbohydrate increases to 135 g/day in the last trimester of pregnancy [19,20]. The recommended dietary allowance (RDA) for dietary carbohydrate in the last trimester pregnancy is up to 175 g/day. The effect of dietary carbohydrate in the last trimester pregnancy remains to be explored. Therefore, it is required to assess whether high carbohydrate intake was associated with vitamin D in pregnant women.

VD deficiency is prevalent in pregnant women worldwide [21,22,23]; however, the prevalence of VD deficiency in Taiwanese pregnant women is unclear. VD is determined by genetic background and acquired endogenously by synthesis in the skin upon exposure to ultraviolet B (UVB) radiation and exogenously from dietary sources and supplementation [24]. Pregnant women in Taiwan usually use sunscreen and extensive clothing cover and work indoors; hence, sun exposure is limited in this group. Furthermore, most pregnant women in Taiwan receive multiple micronutrient (MMN) daily supplements for antenatal care. MMN contains only 200–400 IU of VD instead of a higher dose of VD. The Health Promotion Administration in Taiwan published the 8th edition of the Dietary Reference Intake in 2022, suggesting a daily VD 400 IU for adequate intake (AI) [20], and no extra VD supplementation. However, data on the VD status of Taiwanese pregnant women are limited. The relationship between VD levels and dietary factors among pregnant women also remains unclear in Taiwan. Therefore, we conducted a cross-sectional observation study to investigate the VD status among pregnant women in Taiwan and to determine the dietary, VD supplementation, and anthropometric factors associated with VD levels in these women.

## 2. Materials and Methods

### 2.1. Study Design and Participants

**Figure 1:**
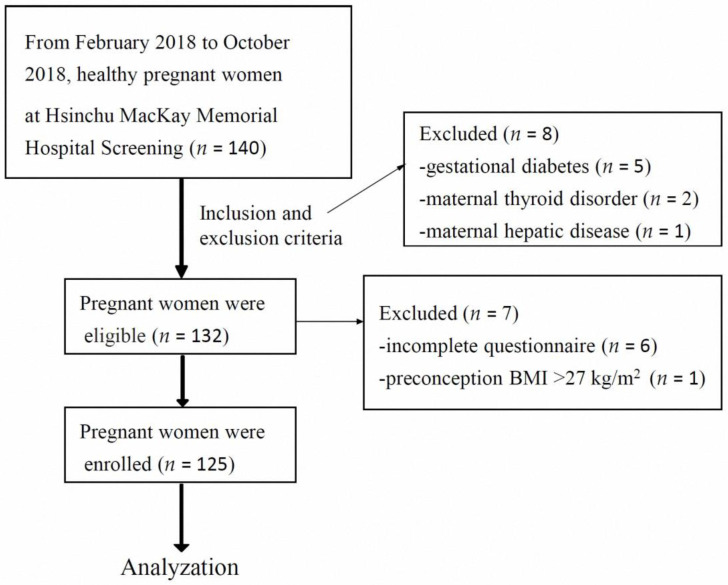
Flowchart of this study. Altogether, 140 pregnant women were recruited; of these, 125 were enrolled and completed the study questionnaire.

This is a prospective cross-sectional observation study to investigate the VD status in Taiwanese pregnant women and the effects of VD supplementation as well as macronutrient intake on serum 25-hydroxy-vitamin D (25[OH]D) level. From February 2018 to October 2018, we conducted a total of 125 healthy pregnant women who had prenatal visits at Hsinchu MacKay Memorial Hospital (Hsinchu City, Taiwan) in this study. The study inclusion criteria were as follows: age ≥ 18 years, average daily outdoor sunlight exposure of <30 min, singleton pregnancy, preconception body mass index (BMI) of <27 kg/m^2^, VD supplementation at a dose of <1000 IU/day during pregnancy, and without systemic and autoimmune diseases. The exclusion criteria included pregnant women with preterm labor, gestational diabetes or diabetes, preeclampsia, oligohydramnios, or polyhydramnios, history of repeated abortion, rheumatoid arthritis, thyroid or parathyroid disorders, hepatic and renal diseases, and history of using aspirin, anticonvulsants, or immunosuppressive drugs. Altogether, 140 pregnant women were recruited; of these, 125 were enrolled and completed the study questionnaire (Figure 1). The Institutional Review Board of MacKay Memorial Hospital approved the study protocol (17MMHIS088e).

### 2.2. Dietary Intake Assessment

An experienced dietitian collected the information on the dietary intake of participants at the hospital using a validated 31-item semi-quantitative food frequency questionnaire (SQ-FFQ) [25]. All data provided by the participants were analyzed to estimate the total energy intake, macronutrient, and cholesterol intake as well as VD supplementation. VD from food was calculated from milk, egg, fish, seafood, organ meat, meat, and vegetable intake. Total VD intake represented the sum of VD from food and VD supplementation. Data on daily sunlight exposure were also obtained. The questionnaire was completed between 30 and 37 weeks’ gestation of pregnant women.

### 2.3. Anthropometric Measurements and Biochemical Analysis

The anthropometric measurements of the pregnant women, including height, weight, BMI, preconception BMI, and weight gain during pregnancy, were performed between 30 and 37 weeks’ gestation. We also assessed the 25(OH)D concentration of pregnant women before delivery. Serum samples were stored at 2–8 °C before assay. The 25(OH)D concentration was measured using chemiluminescence (DiasorinXL, LIAISON; Saluggia, Italy). VD deficiency, insufficiency, and sufficiency were defined as a serum 25(OH)D level of 10–19.9 (25–49.9), 20–29.9 (50–74.9), and 30–100 (75–250) ng/mL (nmol/L), respectively, according to the Endocrine Society guidelines [7]. Severe VD deficiency was defined as a serum 25(OH)D level of <10 ng/mL (<25 nmol/L) [26,27,28].

### 2.4. Statistical Analysis

We analyzed the demographic, VD status, and dietary and anthropometric characteristics of the study participants stratified based on VD status, VD supplementation, and total VD intake. Categorical variables were presented as number or percent, and the between-group differences were analyzed using Fisher’s exact test. Characteristics (continuous variables) were presented as means ± standard deviation and comparisons between groups were tested using *t*-test or one-way analysis of variance (ANOVA). The bivariate Pearson correlation coefficients between the pregnant women 25(OH)D levels and other parameters were assessed. For pregnant women whose carbohydrate intake was ≥300 g/day, Pearson correlation coefficients between 25(OH)D levels and other parameters were assessed and the 25(OH)D levels among groups with different doses of VD supplementation were compared using ANOVA. Multivariate linear regression analysis was performed to analyze the independent factors influencing 25(OH)D levels. Statistical significance was set at *p* < 0.05. All statistical analyses were performed using IBM SPSS Statistics for Windows, version 25 (IBM Corp., Armonk, NY, USA).

Statistically significant difference in 25(OH)D between vitamin D supplementation and no-supplementation groups was detected. The sample size was based on previous study reported by O’Callaghan et al. [29]. At 80% power, α = 0.05, 19 participants in vitamin D supplementation 400 IU/d group and 21 participants in no-supplementation group were required. Assuming dropout, unequal number in each group, and incomplete result for analysis, we enrolled 53 and 45 pregnant women in vitamin D supplementation 400 IU/d group and no-supplementation group, respectively. The power of sample size for the analysis was 91%.

## 3. Results

Altogether, 125 pregnant women were enrolled in this study. Table 1 shows the demographic and dietary intake characteristics of the pregnant women. The participants’ mean age was 32.9 ± 4.4 years (ranging from 20 to 44 years). Sixty-seven (53.6%) and 58 (46.4%) pregnant women were primiparous and multiparous, respectively. The mean 25(OH)D level of the pregnant women before delivery was 17.2 ± 6.8 ng/mL (43 ± 17 nmol/L). Among the 125 enrolled pregnant women, 12.8% (*n* = 16), 55.2% (*n* = 69), 25.6% (*n* = 32), and 6.4% (*n* = 8) had severe VD deficiency, VD deficiency, VD insufficiency, and VD sufficiency, respectively. The proportion of women who did not receive VD supplementation was 36%, whereas 42.4% of the participants received VD supplementation at a dose of 400 IU/day. The average daily sun exposure time was 20.9 ± 12.0 min. Data on the anthropometric characteristics of the pregnant women were also recorded. The participants’ height, weight, BMI, preconception BMI, and weight gain during pregnancy were 160.6 ± 5.3 cm, 67.4 ± 7.8 kg, 26.2 ± 3.0 kg/m^2^, 21.1 ± 2.5 kg/m^2^, and 13.0 ± 3.0 kg, respectively.

The dietary intake of the participants is also shown in Table 1. The average energy intake was 2244 ± 756 kcal/day. For the daily amount of macronutrient intake, the carbohydrate, protein, and fat intake were 335 ± 129, 88.9 ± 31.0, and 59.3 ± 21.4 g, respectively. For the macronutrient intake as daily percentage of energy, the carbohydrate, protein, and fat intake were 59.0 ± 8.4%, 16.2 ± 3.4%, and 24.3 ± 5.6%, respectively. The cholesterol intake was 335 ± 156 mg/day. VD intake from food was 147 ± 97 IU/day. Total VD intake (sum of VD from food and VD supplementation) was 362 ± 206 IU/day.

**Figure 2:**
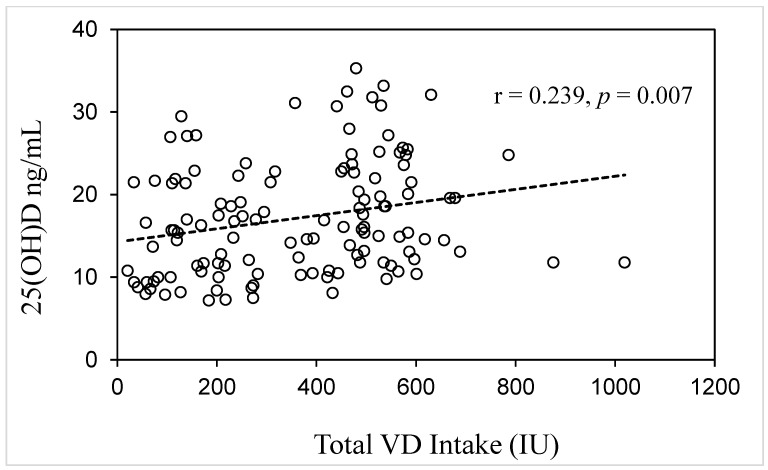
Correlation between serum 25(OH)D level and total VD intake (*n* = 125).

The 25(OH)D level before delivery was significantly correlated with age (r = 0.233, *p* = 0.009) and total VD intake (r = 0.239, *p* = 0.007) (Table 2 and Figure 2), but not with the daily sun exposure time, height, weight, BMI, preconception BMI, weight gain during pregnancy, daily energy intake, macronutrient intake amount and percentage, cholesterol, and VD from food (Table 2). There was no statistically significant difference in the 25(OH)D levels among the seasons.

Among the different VD statuses, there was a significant difference (*p* = 0.041) in the mean age between the severe VD deficiency and VD insufficiency groups (30.2 ± 4.5 years for the severe VD deficient group and 33.9 ± 4.9 years for the VD insufficient group). The severe VD deficiency group had a significantly lower total VD intake (*p* = 0.001) and VD supplementation (*p* < 0.001) than the other groups (Table 2). There were no significant differences in the sun exposure time, total energy intake, macronutrient intake amount and percentage, VD intake from food, anthropometric parameters between the four groups of pregnant women stratified by VD status (Table 3).

The participants in the present study were divided into four groups, i.e., 0, 150, 300 and 400 IU/day VD, based on their VD supplementation. Among the groups stratified based on the different doses of VD supplementation, the 25(OH)D concentration was significantly higher in the VD supplementation 400 IU/d group than in the no-supplementation group (19.2 ± 7.2 ng/mL, *n* = 53 vs. 14.6 ± 6.1 ng/mL, *n* = 45; *p* = 0.007). In the no VD supplementation group, 26.7% of the pregnant women had severe VD deficiency, whereas among the VD supplementation 400 IU/day group, only 3.8% had severe VD deficiency (Table 4). There were no significant differences in sun exposure time, VD from food, and anthropometric parameters across the four groups of pregnant women stratified by VD supplementation (Table 4).

**Figure 3:**
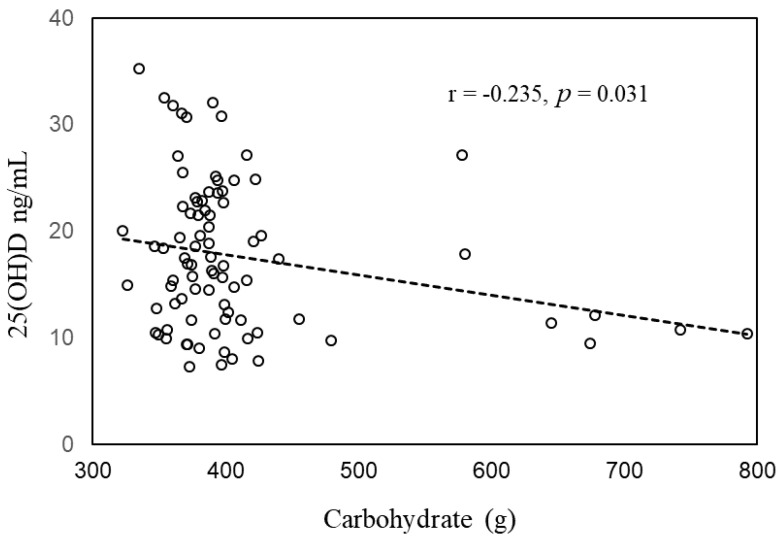
Correlation between serum 25(OH)D level and amount of carbohydrate intake among pregnant women with ≥300 g/day carbohydrate intake (*n* = 85).

Data of 85 pregnant women with a carbohydrate intake of ≥300 g/day were further analyzed. Their 25(OH)D level was significantly correlated with total VD intake (r = 0.366, *p* = 0.001) and significantly negatively correlated with carbohydrate intake (r = −0.235, *p* = 0.031) (Figure 3). There were 40 pregnant women with a carbohydrate intake of <300 g/day were also evaluated, the 25(OH)D level was not significantly correlated with carbohydrate intake (r = −0.048, *p* = 0.770). The 25(OH)D concentration was significantly higher in the VD supplementation group receiving 400 IU/day than in the no-supplementation group (20.0 ± 7.2 ng/mL vs. 13.9 ± 5.9 ng/mL, *p* = 0.003). With the 25(OH)D level as the dependent variable, and carbohydrate intake, total VD intake, and VD supplementation of 400 IU/day as independent variables, the results of the multiple linear regression analysis suggested that carbohydrate intake (β = −0.236, *p* = 0.022) was independently and negatively correlated with serum 25(OH)D levels, and total VD intake was independently and positively correlated with serum 25(OH)D levels (β = 0.377, *p* = 0.021) (Table 5).

## 4. Discussion

Our study showed that the mean 25(OH)D level of the enrolled pregnant women was 43 nmol/L or 17.2 ng/mL. More than two-thirds of the 125 enrolled pregnant women presented with VD deficiency or severe VD deficiency. The high prevalence of VD deficiency and insufficiency among pregnant women in this study is similar to those of studies conducted in Switzerland in 2012–2015, China in 2016, Japanese in 2019, and Napel in 2020 [30,31,32,33]. Swiss women had a mean serum 25(OH)D level of approximately 37 nmol/L (15 ng/mL) and one-third of the overall study population (*n* = 1382) had a serum 25(OH)D level < 25 nmol/L (<10 ng/mL); thus, they were seriously vitamin D deficient [30]. Kanatani et al. showed that 73.2% of Japanese pregnant women had 25(OH)D levels of <20 ng/mL (<50 nmol/L) [33]. An Indonesian observational study in 2018 showed that 61.25% of pregnant women had VD deficiency or insufficiency; VD deficiency was still prevalent in this sunny country if no VD supplementation was provided [34]. Bodnar et al. showed that VD deficiency and insufficiency occurred in 83.3% of black women at delivery and 47.1% of white women, respectively [14]. Our study showed that the 25(OH)D levels significantly correlated with total VD intake but not with VD from food. Our results also showed that the group with regular VD supplementation at a dose of 400 IU/day had higher serum 25(OH)D levels than the no-supplementation group. However, >60% of the participants still had severe VD deficiency or VD deficiency in this group. This result is consistent with those of previous studies, and the importance of VD supplementation in pregnant women is reinforced. Supplementation with a higher dose of VD may be needed to improve the VD status of pregnant women.

The recommended dose of VD intake and supplementation remains controversial. According to the Dietary Reference Intakes “8th Edition” in Taiwan in 2020, this study showed that the participants had higher dietary energy intake and acceptable macronutrient distribution ranges and amount of protein intake. However, higher carbohydrate intake above the recommended dietary allowance (RDA) and lower VD intake than the AI were also noted. The AI of VD among pregnant women is 400 IU/day in Taiwan [20]. In the present study, those whose total VD intake was ≥400 IU/day had higher 25(OH)D levels and were more likely VD sufficient. Furthermore, we observed a higher VD intake from food and higher protein intake in those with a total VD intake of ≥400 IU/day. However, despite the total VD intake of ≥400 IU/day, the pregnant women in the present study still had low 25(OH)D level and 59% of them had VD deficiency. Current recommendations worldwide show no consensus about the amount of acceptable VD intake during pregnancy. According to a lot of respective meta-analyses [8,35,36], the conclusion is that there is no universal agreement regarding the appropriate dose and the time of commencement of VD supplementation during pregnancy. The RDA from IOM (U.S.) is 600 IU/day of VD [19,37]. The Endocrine Society and American College of Obstetricians and Gynecologists recommended that the VD intake of 1000–2000 IU/day is safe for pregnant mothers with VD deficiency [7,38]. In 2020, the World Health Organization recommended that pregnant women with suspected VD deficiency, VD supplements may be given at a dose of 200 IU/day [39]. Therefore, further studies are required to determine the optimal VD supplementation strategy for pregnant women. However, the present study has revealed a high prevalence of Taiwanese pregnant women without VD supplementation. To maintain VD sufficiency, adequate nutrition rich in VD, sufficient sun exposure, and regular VD supplementation should be recommended for pregnant women.

Pregnant women were reported to have sub-optimal carbohydrate and VD knowledge, and less than half of them had received nutrition education during pregnancy [40]. In our study, carbohydrate intake was significantly negatively correlated with 25(OH)D levels in the group with high carbohydrate intake, i.e., ≥300 g/day carbohydrate intake which was based on two-fold between EAR and RDA from IOM (U.S.) and Taiwan’s Dietary Reference. Only a few studies have addressed the relationship between VD and high carbohydrate intake [17,18]. Bolesławska et al. reported that the 25(OH)D concentration was significantly higher in the low-carbohydrate-high-fat diet group than in the Eastern European diet group [41]. Alathari et al. also showed that individuals consuming a low carbohydrate diet (<62%) and those having a low genetic metabolic risk had significantly higher levels of 25(OH)D [42]. A recent study in 2021 found that women with an increased genetic risk of VD deficiency and who consume higher amounts of carbohydrates had increased body fat composition [18]. Although the mechanism between dietary carbohydrate intake and serum 25(OH)D concentration is still unclear. Increased body fat tissue may exert volumetric dilution, VD sequestration, and impaired hepatic 25-hydroxylation, and further cause VD deficiency [43].

This study has several strengths. This was the first study to investigate the VD status and dietary factors among pregnant women with limited sun exposure time and low dose VD supplementation in Taiwan. Data on daily sun exposure time and amount of VD supplementation, as important factors for VD status, were also collected. The validated SQ-FFQ administered by an experienced dietitian increases the accuracy of dietary intake assessment. However, this study had several limitations. The SQ-FFQ can only obtain self-reported dietary intake information from the participants, and the 31-item questionnaire did not include all the VD food consumed in Taiwan. The estimates of VD intake may not be accurate. Furthermore, although the amount of carbohydrate intake may be related to the VD status in the present study, the SQ-FFQ did not include the total carbohydrate count and did not quantify different classes of carbohydrates. Moreover, the metabolic parameters, calcium metabolism, and body fat among pregnant women were not investigated. Since VD supplementation may be an important factor for achieving an acceptable VD status among pregnant women, only women with VD supplementation of ≤400 IU/day were included in this study. The effect of higher dose VD supplementation was not assessed. Finally, the major limitation of this study was the relatively small number of participants. Hence, further studies with larger populations should be conducted in the future.

## 5. Conclusions

VD deficiency is widespread among pregnant women in Taiwan. Over two-thirds of pregnant women present with VD deficiency or severe VD deficiency. Total VD intake is a major determinant of VD status among pregnant women. VD supplementation increases the 25(OH)D concentration as compared with no-supplementation. Higher carbohydrate intake (≥300 g/day) may negatively affect VD status of the pregnant women. Moreover, the current VD dietary intake recommendations are inadequate to meet the demands of pregnancy. Supplementation with a higher dose of VD is needed to improve the VD status of pregnant women.

## Figures and Tables

**Table 1:** Demographic characteristics and dietary intake details of the participants.

	Pregnant Women *n* = 125
Age (years)	32.9 ± 4.4
Primiparous, *n* (%)	67 (53.6)
Multiparous, *n* (%)	58 (46.4)
Height (cm)	160.6 ± 5.3
Weight (kg)	67.4 ± 7.8
Preconception BMI (kg/m^2^)	21.1 ± 2.5
BMI (kg/m^2^)	26.2 ± 3.0
Weight gain during pregnancy (kg)	13.0 ± 3.0
25(OH)D (ng/mL)	17.2 ± 6.8
Severe VD deficiency, *n* (%)	16 (12.8)
VD deficiency, *n* (%)	69 (55.2)
VD insufficiency, *n* (%)	32 (25.6)
VD sufficiency, *n* (%)	8 (6.4)
Vitamin D supplementation (IU/day)	214 ± 180
0 IU/day	45 (36.0)
150 IU/day	17 (13.6)
300 IU/day	10 (8.0)
400 IU/day	53 (42.4)
Duration of daily sun exposure (min)	20.9 ± 12.0
Daily energy intake (kcal/day)	2244 ± 756
Total carbohydrate (g)	335 ± 129
Total protein (g)	88.9 ± 31.0
Total fat (g)	59.3 ± 21.4
Carbohydrate (% of energy)	59.0 ± 8.4
Protein (% of energy)	16.2 ± 3.4
Fat (% of energy)	24.3 ± 5.6
Cholesterol (mg)	335 ± 156
VD from food (IU/day)	147 ± 97
Total VD intake (IU/day)	362 ± 206

Values are means ± standard deviation or numbers (percent). Abbreviations: VD, vitamin D; BMI, body mass index.

**Table 2:** Correlation of 25(OH)D levels before delivery with study variables.

Variables	25(OH)D Pearson Coefficient r	*p* Value
Age	0.233	0.009
Duration of daily sun exposure	0.071	0.433
Height	0.069	0.445
Weight	−0.105	0.242
BMI	−0.139	0.122
Preconception BMI	−0.076	0.399
Weight gain during pregnancy	−0.125	0.164
Daily energy intake	−0.040	0.656
Total carbohydrate	−0.023	0.802
Total protein	−0.050	0.583
Total fat	−0.071	0.430
Carbohydrate (% of energy)	0.059	0.516
Protein (% of energy)	−0.054	0.549
Fat (% of energy)	−0.073	0.421
Cholesterol	−0.070	0.436
VD from food	−0.054	0.549
Total VD intake	0.239	0.007

Abbreviations: BMI, body mass index; VD, vitamin D.

**Table 3:** Demographic, dietary, and anthropometric characteristics and vitamin D levels of the study participants stratified based on vitamin D status.

Characteristics	Severe VD Deficiency (*n* = 16) ^a^	VD Deficiency (*n* = 69) ^b^	VD Insufficiency (*n* = 32) ^c^	VD Sufficiency (*n* = 8) ^d^	*p* Value
Age (years)	30.2 ± 4.5	32.9 ± 4.2	33.9 ± 4.9	33.8 ± 1.7	0.041 c > a
25(OH)D (ng/mL)	8.5 ± 0.8	14.3 ± 3.0	23.9 ± 2.4	32.2 ± 1.5	<0.001 d > c > b > a
Duration of daily sun exposure (min)	19.7 ±13.2	21.1 ±12.1	20.2 ±12.5	24.4 ± 8.2	0.813
Total energy intake (kcal/day)	2145 ± 807	2307 ± 830	2181 ± 630	2150 ± 443	0.784
Carbohydrate (g/day)	330 ± 150	340 ± 139	327 ± 107	337 ± 88	0.968
Protein (g/day)	82.8 ±33.0	92.3 ±33.0	86.9± 28.0	79.4 ±19.7	0.516
Fat (g/day)	53.8 ± 19.3	62.6 ±23.9	56.9 ±17.1	52.0 ±14.8	0.264
Carbohydrate (%)	59.9 ±10.5	58.2 ±8.3	59.5 ±7.1	61.8 ±10.5	0.629
Protein (%)	16.1 ± 4.8	16.4 ± 3.3	16.0 ± 2.8	15.0 ± 3.5	0.766
Fat (%)	23.8 ± 6.7	24.9 ± 5.5	23.9 ± 4.9	22.4 ± 7.6	0.577
Cholesterol (mg)	299 ± 152	359 ± 171	320 ± 127	258 ± 102	0.190
VD from food (IU/day)	115 ± 79	159 ± 106	146 ± 88	105 ± 61	0.236
VDs (IU/day)	68 ± 138	221 ± 178	226 ± 177	387± 35	<0.001 a < b, c, d & b < d
VDs, 0 IU/day	12 (75)	23 (33.3)	10 (31.3)	0	
VDs, 150 IU/day	2 (12.5)	10 (14.5)	5 (15.6)	0	
VDs, 300 IU/day	0 (0)	6 (8.7)	3 (9.4)	1 (12.5)	
VDs, 400 IU/day	2 (12.5)	30 (43.5)	14 (43.8)	7 (82.5)	
Total VD intake (IU/day)	184 ± 147	382 ± 207	374 ± 206	493 ± 79	0.001 a < b, c, d
Height (cm)	159.9 ± 4.8	160.4 ± 5.5	161.0 ± 5.2	161.6 ± 5.2	0.847
Weight (kg)	67.2 ± 6.8	68.0 ± 8.5	66.5 ± 7.2	66.9 ± 5.8	0.850
BMI (kg/m^2^)	26.3 ± 2.5	26.4 ± 3.2	25.7 ± 3.0	25.6 ± 2.4	0.699
Preconception BMI (kg/m^2^)	21.5 ± 2.0	21.2 ± 2.4	20.8 ± 2.9	21.3 ± 2.8	0.790
Weight gain during pregnancy (kg)	12.3 ± 3.1	13.4± 5.2	11.3 ± 1.6	13.0 ± 4.3	0.526

Values are means ± standard deviation or numbers (%). Abbreviations: VD, vitamin D; VDs, vitamin D supplementation; BMI, body mass index. a = severe VD Deficiency group, b = VD deficiency group, c = VD Insufficiency group, d = VD Sufficiency group.

**Table 4:** Demographic, vitamin D status, dietary and anthropometric characteristics of the study participants stratified based on vitamin D supplementation.

Characteristics	0 IU/Day (*n* = 45) ^a^	150 IU/Day (*n* = 17) ^b^	300 IU/Day (*n* = 10) ^c^	400 IU/Day (*n* = 53) ^d^	*p* Value
Age (years)	32.2 ± 4.8	32.2 ± 3.8	37 ± 3.7	32.9 ± 4.1	0.015 c > a, b, d
25(OH)D (ng/mL)	14.6 ± 6.1	16.9 ± 5.3	18.3 ± 7.2	19.2 ± 7.2	0.007 d > a
Severe VD deficiency, *n* (%)	12 (26.7)	2 (11.8)	0 (0)	2 (3.8)	
VD deficiency, *n* (%)	23 (51.1)	10 (58.8)	6 (60)	30 (56.6)	
VD insufficiency, *n* (%)	10 (22.2)	5 (29.4)	3 (30)	14 (26.4)	
VD sufficiency, *n* (%)	0 (0)	0 (0)	1 (10)	7 (13.2)	
Duration of daily sun exposure (min)	21.4 ± 11.4	17.9 ±12.6	26.0 ±9.7	20.4 ± 12.7	0.394
Total VD intake (IU/day)	151 ± 92	276 ± 89	442 ± 72	553 ± 108	<0.001 d > c > b > a
VD from food (IU/day)	148 ± 92	126 ± 89	142.± 72	153 ± 108	0.799
Height (cm)	161.6 ± 5.5	160.2 ± 5.9	158.5 ± 3.3	160.2 ± 5.1	0.328
Weight (kg)	68.9 ± 8.1	66.3 ± 6.2	66.9 ± 9.1	66.6 ± 7.8	0.466
BMI (kg/m^2^)	26.4 ± 3.2	25.9 ± 2.7	26.6 ± 3.1	26.0 ± 3.1	0.842
Preconception BMI (kg/m^2^)	21.5 ± 2.6	21.6 ± 2.4	21.2 ± 2.0	20.7 ± 2.5	0.409
Weight gain during pregnancy (kg)	12.9 ± 4.0	11.1± 3.7	13.5 ± 4.4	13.5 ± 4.7	0.251

Values are means ± standard deviation or number (%). Abbreviations: VD, vitamin D; BMI, body mass index. a = no VD supplementation group, b = VD supplementation 150 IU/d group, c = VD supplementation 300 IU/d group, d = VD supplementation 400 IU/d group.

**Table 5:** Multiple linear regression analysis of predictive factors for 25(OH)D levels in subjects with ≥300 g/day carbohydrate intake.

Variables	B	β	*p* Value
Carbohydrate intake	−0.019	−0.236	0.022
Total VD intake	0.014	0.377	0.021
VDs (400 IU/day)	−0.200	−0.014	0.929

Abbreviations: VD, vitamin D; VDs, vitamin D supplementation.

## Data Availability

The data that support the findings of this study are available from the corresponding author upon reasonable request.
